# Editorial for the Special Issue “Management, Development, and Breeding of *Coffea* sp. Crop”

**DOI:** 10.3390/plants15162455

**Published:** 2026-08-12

**Authors:** Weverton Pereira Rodrigues, Fábio Luiz Partelli

**Affiliations:** 1 Centro de Ciências Agrárias, Universidade Estadual da Região Tocantina do Maranhão, Imperatriz 65900-001, MA, Brazil; 2 Centro Universitário Norte do Espírito Santo, Universidade Federal do Espírito Santo, São Mateus 29932-540, ES, Brazil

Coffee cultivation is far more than the production of a globally traded beverage. Across many tropical regions, it structures rural landscapes, supports family farming, creates employment, stimulates local commerce, and generates income throughout a broad network that includes nurseries, farms, cooperatives, processors, exporters, roasters, and service providers. Its capacity to circulate value within producing territories makes coffee an important driver of local and regional development and, in several countries, a strategic component of agricultural exports and national economic activity. The crop also has a strong social dimension, i.e., when supported by science, technology transfer, and organized value chains, coffee production can strengthen livelihoods, encourage permanence in rural areas, and promote social transformation in the countryside.

The relevance of coffee is especially visible in territories where a large proportion of production is carried out by small and medium-sized growers. In these settings, improvements in yield, crop stability, disease resistance, and production efficiency can have effects that extend beyond the farm gate. They can increase household income, create opportunities for rural employment, strengthen cooperatives and local enterprises, and improve the capacity of communities to invest in education, infrastructure, and value-added activities. Consequently, advances in coffee science should be understood not only as technical achievements, but also as instruments capable of supporting territorial development and reducing the vulnerability of farming families to climatic, biological, and economic shocks.

This contribution of coffee to development, however, depends on the biological and economic sustainability of production systems. Climate change, increasingly irregular rainfall, thermal extremes, pests and diseases, limited genetic diversity in some cultivated populations, and fluctuations in yield and market prices impose interconnected challenges. These pressures cannot be addressed through isolated interventions. They require an integrated understanding of the coffee production chain, from plant development and genetic improvement to crop management, yield formation, plant health, and economic return. The articles published in this Special Issue reflect this broad perspective and demonstrate how complementary fields of research can converge to improve the resilience, productivity, and long-term viability of coffee cultivation.

A logical starting point for this integrated view is the reproductive cycle itself. Unigarro et al. [1] provide a comprehensive review of flowering and fruiting in *Coffea arabica*, organizing the reproductive process through a phenological perspective informed by the extended BBCH scale. The review connects flower and fruit morphology, developmental phases, environmental regulation, and competition for assimilates between vegetative and reproductive sinks. This synthesis is particularly relevant because the timing and intensity of flowering and fruit growth determine not only yield formation but also the scheduling of irrigation, fertilization, pest management, and harvest operations. By emphasizing that phenology is shaped by both biotic and abiotic factors, the authors offer a framework for aligning management decisions with the actual developmental stage of the crop. Such alignment is essential under increasingly variable climatic conditions, in which traditional calendar-based recommendations may become less reliable.

The capacity to adapt coffee production to changing environments begins with the genetic resources available to breeding programs. Silva et al. [2] characterized cultivated *Coffea canephora* genotypes from Rondônia, in the Brazilian Amazon, using SNP markers and molecular markers linked to resistance to coffee leaf rust and coffee berry disease. The analysis revealed five major genetic groups, including materials predominantly associated with Robusta, Conilon, and intervarietal hybrid backgrounds. Importantly, three genotypes carried a complete pyramided configuration of resistance alleles for both diseases, while several others contained different combinations of resistance loci. Beyond clarifying the genetic structure, the marker profiles contributed to the classification and traceability of genotypes according to origin, botanical variety, and genealogy. These results show how molecular characterization can guide parental selection, increase the efficiency of crosses, and support the development of disease-resistant and climate-resilient clones and hybrids. Although molecular markers cannot replace field validation, they reduce uncertainty and help breeding programs use diversity more strategically.

Accurate phenotyping is the necessary counterpart to molecular information. Unigarro et al. [3] evaluated non-destructive approaches for estimating leaf size and shape in 55 advanced *C. arabica* progenies derived from intra- and interspecific crosses. The Montgomery model provided reliable estimates for individual progenies, hybridization types, and pooled datasets, with a consistent coefficient of approximately 0.67. In contrast, the model based on the principle of similarity was suitable mainly when fitted separately for each progeny. The study also demonstrated that the width-to-length ratio, ellipticity, and leaf size influence model parameters. These findings are methodologically important because leaf area is closely related to radiation interception, transpiration, photosynthetic capacity, and crop growth. A rapid and inexpensive non-destructive method allows for repeated measurements on the same leaves and plants, supporting longitudinal studies, high-throughput phenotyping, and more efficient selection in breeding populations.

Phenotypic expression, however, cannot be interpreted independently of the environment. Silva et al. [4] investigated leaf anatomical responses of *Coffea* spp. genotypes under full-sun monoculture at low and high altitudes and under a low-altitude agroforestry system, in winter and summer. The genotype affected all of the anatomical traits evaluated, while genotype × environment interactions accounted for a substantial proportion of the variation in key traits. Management effects were generally stronger than seasonal effects for several traits, and the agroforestry system altered leaf anatomy more markedly than the difference in altitude between the full-sun sites. Under shade, leaves showed reduced palisade and spongy parenchyma thickness, lower total leaf thickness and stomatal density, and thicker epidermal layers. The results highlight the plasticity of coffee leaves and demonstrate that anatomical traits emerge from the interaction between genetic constitution, management, and season. This knowledge can support the identification of genotypes suited to specific production systems and reinforce the role of agroforestry as a distinct environment rather than merely a version of full-sun cultivation with less radiation.

Environmental adaptation also involves the management of thermal stress at the boundaries of coffee cultivation. Suganami et al. [5] examined whether root-zone heating could mitigate cold injury in *C. arabica* exposed to low air temperatures. Heating the root zone maintained the leaf relative water content above 90%, whereas non-heated plants declined to approximately 70% and exhibited wilting. Defoliation exceeded 50% in the control but was reduced by about 20% under root-zone heating. Photosynthesis fell sharply during cold exposure in both treatments; nevertheless, surviving leaves in heated plants recovered photochemical efficiency and regained more than half of their pre-treatment CO_2_ assimilation and electron transport rates within one week. The study indicates that maintaining root water uptake can reduce the hydraulic imbalance caused by cold and dry air, preserve foliage, and accelerate post-stress recovery. Therefore, localized root-zone heating may offer a more energy-efficient alternative to heating an entire greenhouse, expanding the possibilities for coffee cultivation in temperate or marginal environments.

Biotic stress is another major constraint, the intensity of which may increase as the climate changes. Molina et al. [6] evaluated F3 progenies derived from crosses between the agronomically adapted and rust-resistant Castillo^®^ variety and Ethiopian *C. arabica* introductions with antibiosis to *Hypothenemus hampei*. Thirteen progenies combined medium stature, high productivity, rust resistance, and reduced coffee berry borer development. Oviposition was reduced by 18.0–25.8% under controlled conditions and by 24.1–69.8% in field evaluations compared with the susceptible control. Simulations under Neutral and El Niño scenarios suggested that progenies reducing oviposition by 34–55% could maintain infestation below the 5% economic damage threshold during the eight-month fruit development period. This work is especially significant because it integrates conventional breeding, host plant resistance, field validation, and climate-based modeling. Antibiosis does not eliminate the need for integrated pest management, but it can reduce dependence on insecticides, delay population growth, and make control programs more robust under warmer conditions favorable to the pest.

Ultimately, scientific advances must translate into stable production and improved economic outcomes for growers. Kolln et al. [7] integrated multi-year yield stability and gross revenue in the evaluation of 28 *C. canephora* genotypes grown in Rondônia over five harvest seasons. The trial achieved a cumulative mean of 306.22 bags ha^−1^, equivalent to 61.24 bags ha^−1^ per harvest, but genotypes differed markedly in temporal yield patterns. BAG19, BRS1216, GJ8, GJ25, AS2, and BAG24 were closest to the ideotype combining maximum yield and stability. Across contrasting historical price scenarios, these genotypes also showed superior gross revenue. Their mean yield was 85% higher and their mean gross revenue 61.2% higher than the overall mean of the 28 genotypes. Notably, differences in yield potential contributed more to revenue variation than biennial bearing. By combining biological performance and economic analysis, the study provides a decision-oriented approach to clone recommendation and demonstrates that productive stability should be considered together with profitability, not as an isolated breeding statistic.

Another important feature of this collection is the connection between different scales of analysis. Some studies focus on genes, alleles, and molecular diversity, whereas others examine leaves, whole-plant physiology, reproductive development, pest interactions, multi-year productivity, and farm-level revenue. Considered separately, each scale answers a specific question; considered together, they show how coffee performance emerges from a chain of biological processes and management decisions. This multiscale perspective is particularly valuable for a perennial crop, in which the consequences of cultivar choice and management may persist for many years and where the cost of correcting an unsuitable decision can be high.

Taken together, the contributions in this Special Issue describe a continuum from basic plant development to farm-level economic return. Phenology defines the temporal organization of the production cycle [1]; molecular diversity and resistance-gene profiling expand the options available to breeders [2]; non-destructive phenotyping improves the measurement of plant traits [3]; anatomical studies reveal how genotypes respond to management and season [4]; localized heating offers a strategy to mitigate cold stress [5]; antibiosis-based breeding strengthens coffee berry borer management under climate change [6]; and the integration of yield stability with gross revenue connects genetic performance to producers’ economic security [7].

This collective evidence reinforces the central message that the sustainability of coffee cultivation depends on integration. Genetic improvement must be connected to physiology and phenology, management must account for genotype-specific responses, pest and disease resistance must be evaluated under future climate scenarios, and agronomic superiority must be translated into stable income. Research organized around this systems perspective can help coffee-growing regions remain productive, competitive, and socially dynamic while responding to environmental uncertainty. The studies assembled here contribute tools, knowledge, and conceptual bridges for that task, and they also identify future priorities, including multi-environment validation, long-term field assessment, high-throughput phenotyping, integrated modeling, and closer links between biological innovation and the realities of farmers and coffee value chains. In practical terms, this means that cultivar development, crop management, and economic evaluation should be planned as connected components of the same innovation process. It also means that recommendations must be tested across contrasting environments and production systems so that scientific progress can be converted into reliable solutions for growers.

## Data Availability

All papers cited in this Editorial are available via the following link: https://www.mdpi.com/journal/plants/special_issues/FG19698AKT (accessed on 28 July 2026).
